# Ilizarov technology in China: a historic review of thirty-one years

**DOI:** 10.1007/s00264-021-05238-4

**Published:** 2021-10-13

**Authors:** Yue-Liang Zhu, Bao-Feng Guo, Jian-Chen Zang, Qi Pan, Ding-Wei Zhang, Yue Peng, Si-He Qin

**Affiliations:** 1 Department of Orthopedic Surgery, 2nd Affiliated Hospital, School of Medicine, Zhejiang University, 88#, Jiefang Road, Hangzhou, 310009 China; 2 Department of Orthopedic Surgery, ChuiYangLiu Hospital Affiliated To Tsinghua University, Beijing, China; 3 Center for Bioethics, School of Population Medicine and Public Health, Chinese Academy of Medical Sciences/ Peking Union Medical College, Beijing, China; 4 Department of Pediatric Orthopedics, Pinghu Hospital, Health Science Center, Shenzhen University, Shenzhen, China; 5 Department of Orthopedic Surgery, Central Hospital of Mianyang, Mianyang, China; 6 Department of Orthopedic Surgery, Peking Union Medical College Hospital, Chinese Academy of Medical Sciences and Peking Union Medical College, Beijing, China; 7 Rehabilitation Hospital, National Research Center for Rehabilitation Technical Aids; Key Laboratory of Human Motion Analysis and Rehabilitation Technology of the Ministry of Civil Affairs, Beijing, China

**Keywords:** Ilizarov technology, Orthopedic heritage, External Fixation, China, Modifications

## Abstract

**Purpose:**

To summarize the evolution of Ilizarov technology in China, highlight important milestones, introduce the atmosphere of the era concerning the first uses and development of this technology, and share Chinese modification and experience in this field.

**Method:**

A thorough interview with senior ASAMI members of China and literature search and physical books in libraries was undertaken to summarize the history of Ilizarov technology in China.

**Results:**

The formal development of Ilizarov technology began when professor Ilizarov himself came to Beijing (1991) and gave a speech. In the following 31 years, his technology was rapidly developed through China, with many symposiums held and associations established including ASAMI China (2003) and ILLRS China (2015). Today, Ilizarov technology has become the main treatment of complex fractures, defects, nonunion, infections, deformities, and chronic ischemic ulcers of the limbs. In those years, Chinese scholars also developed some special treatment methods and made many modifications to Ilizarov external fixators.

**Conclusion:**

Ilizarov technology has developed in China for 31 years. It revolutionized the treatment of complex limb traumas, deformities, and diseases. In the treatment of millions of patients, Chinese scholars had many unique experiences and made modifications to this technology which is worthy to share with the world.

## Introduction

**Fig. 1:**
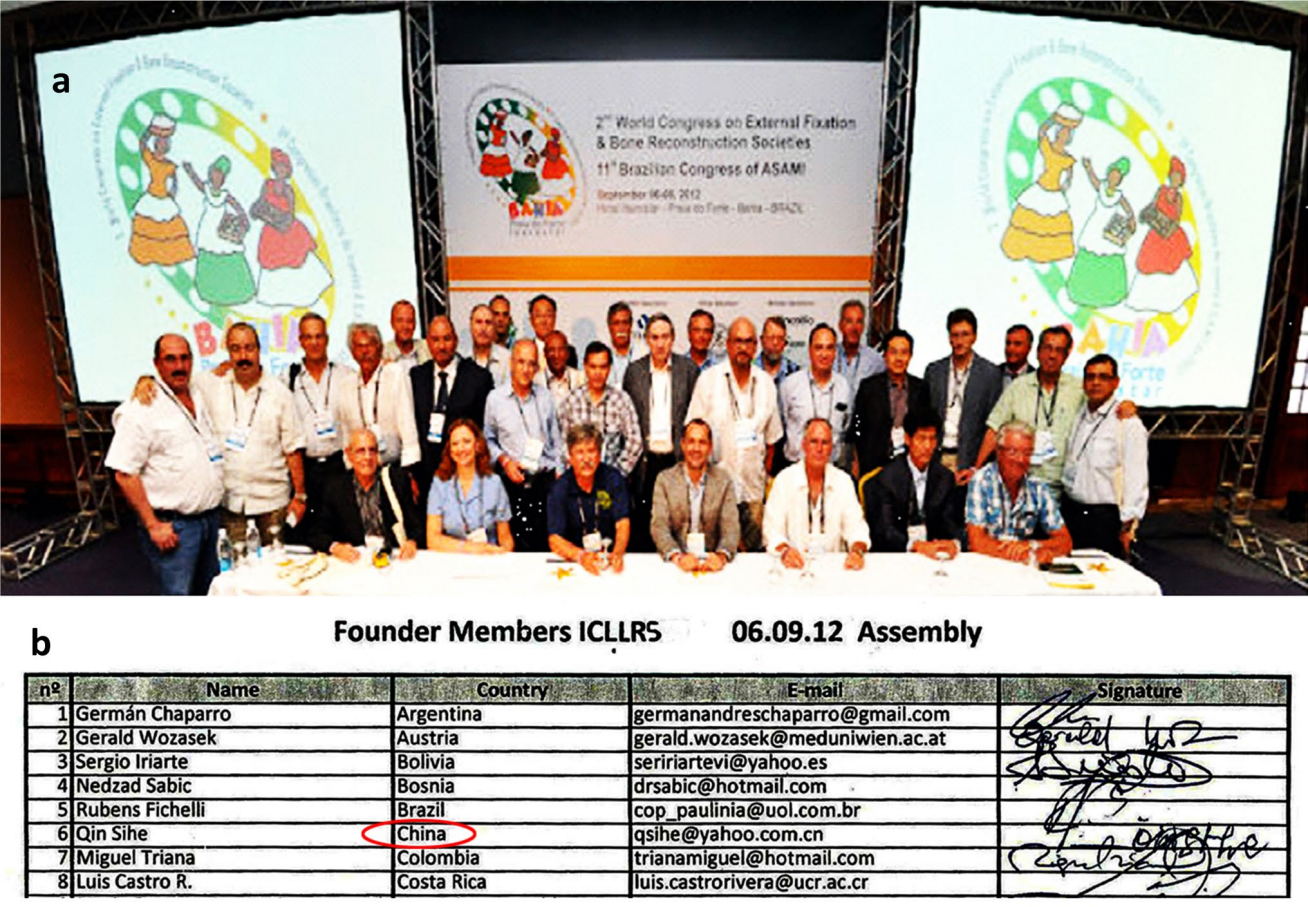
ILLRS in Brazil, 2012 (**a**), China as a full member (**b**)

In 2003, with the support of the Chinese Journal of Orthopedics, the first Ilizarov Technology Symposium was held in Beijing, attended by 120 orthopaedic doctors from 23 provinces and regions. The symposium established ASAMI China, with Si-He Qin as the first chairman, marking a new stage of Ilizarov technology development in China. Every year, ASAMI China organizes various Ilizarov technology courses in China. In 2012, *the Second World Congress of External Lengthening and Bone Reconstruction* was held in Brazil. Professor Nuno initiated the establishment of the International Limb Lengthening and Reconstruction Society (ILLRS). Qin signed on behalf of China as a full member of ILLRS (Fig. 1). Then, in 2015, ILLRS China was established.

Based on millions of surgical procedures such as serious trauma, poliomyelitis sequelae, cerebral palsy, and other congenital diseases, Ilizarov technology used in China differs from Russia and the Western world in many aspects—the instruments and techniques were modified accordingly [1–3]. On the occasion of the 100th anniversary of the birth of Ilizarov, it is worthy to review the history of Ilizarov technology in China and share our experience with the world.

The development and application of external fixation technology in China began in the mid-1970s.

## Pioneers and leading proponents

**Fig. 2:**
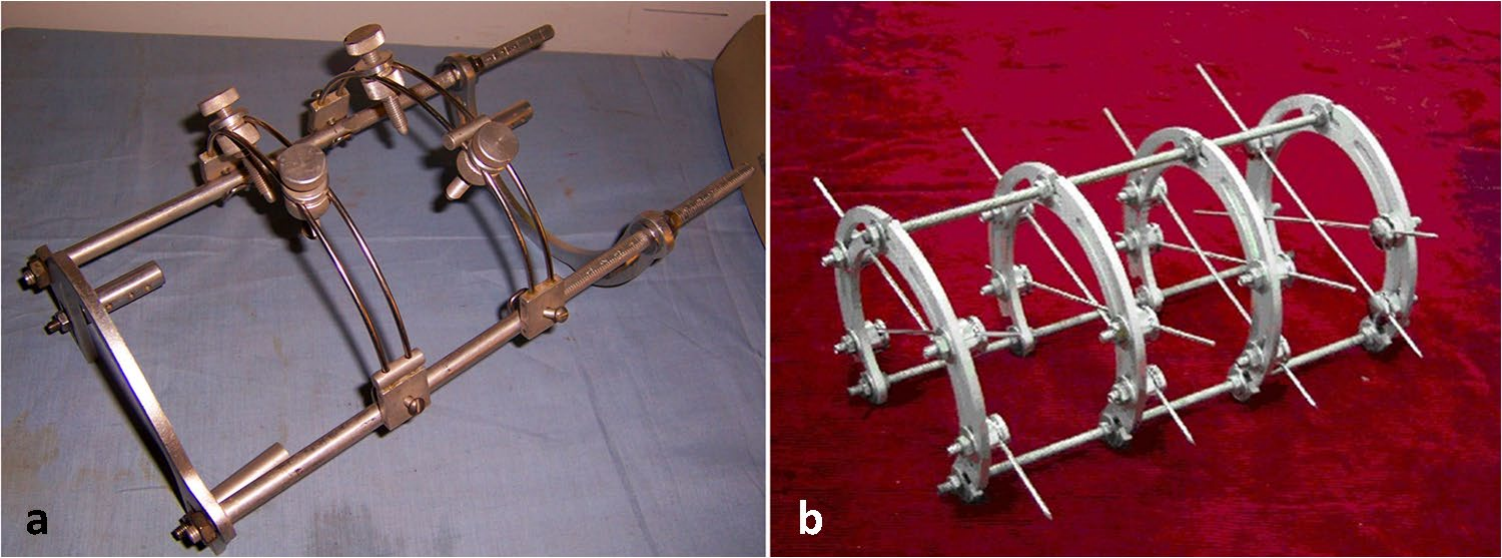
He Meng’s external fixator (**a**) and Qi-Hong Li’s half-ring groove external fixation (**b**)

In 1976, the Tangshan earthquake (7.83 on the Richter scale) occurred. In order to quickly treat hundreds of thousands of patients with traumatic fracture, He Meng invented an external fixator on the basis of Chinese traditional medicine theory in which the fractures were manually reduced and fixed with min-splints of bark (Fig. 2). Meng published the book *Chinese Fracture Reduction and Fixation Therapy* [4]. At that time, no Chinese ever heard Ilizarov external fixation.

In 1980, Qi-Hong Li went to the Soviet Union and got a fellowship in Central Institute of Traumatology and Orthopaedics. He returned in 2 years and devised a half-ring groove external fixator for the animal experiment of distraction osteogenesis (Fig. 2) [5, 6]. In 1994, he initiated the establishment of the *Chinese Society of External Fixation* which laid the foundation for the later academic work of Ilizarov technology.

In 1988, Quan-Mu Chen of Taipei Veterans General Hospital visited Kurgan of Russia and firstly used Ilizarov techniques in Taiwan.

In 1989, Si-He Qin invited Vasilyevich, director of the Ilizarov technology center in the Far East of Russia (Krisnikov) to Harbin. This is the first time the mainland surgeons saw Ilizarov techniques. Shao-Chuan Pan of Beijing Children’s Hospital visited Scottish Rite Children’s Hospital (US) and learned Ilizarov techniques under Professor Birch. When he returned, he immediately wrote a paper to introduce Ilizarov techniques in the *Chinese Journal of Surgery* [7].

**Fig. 3:**
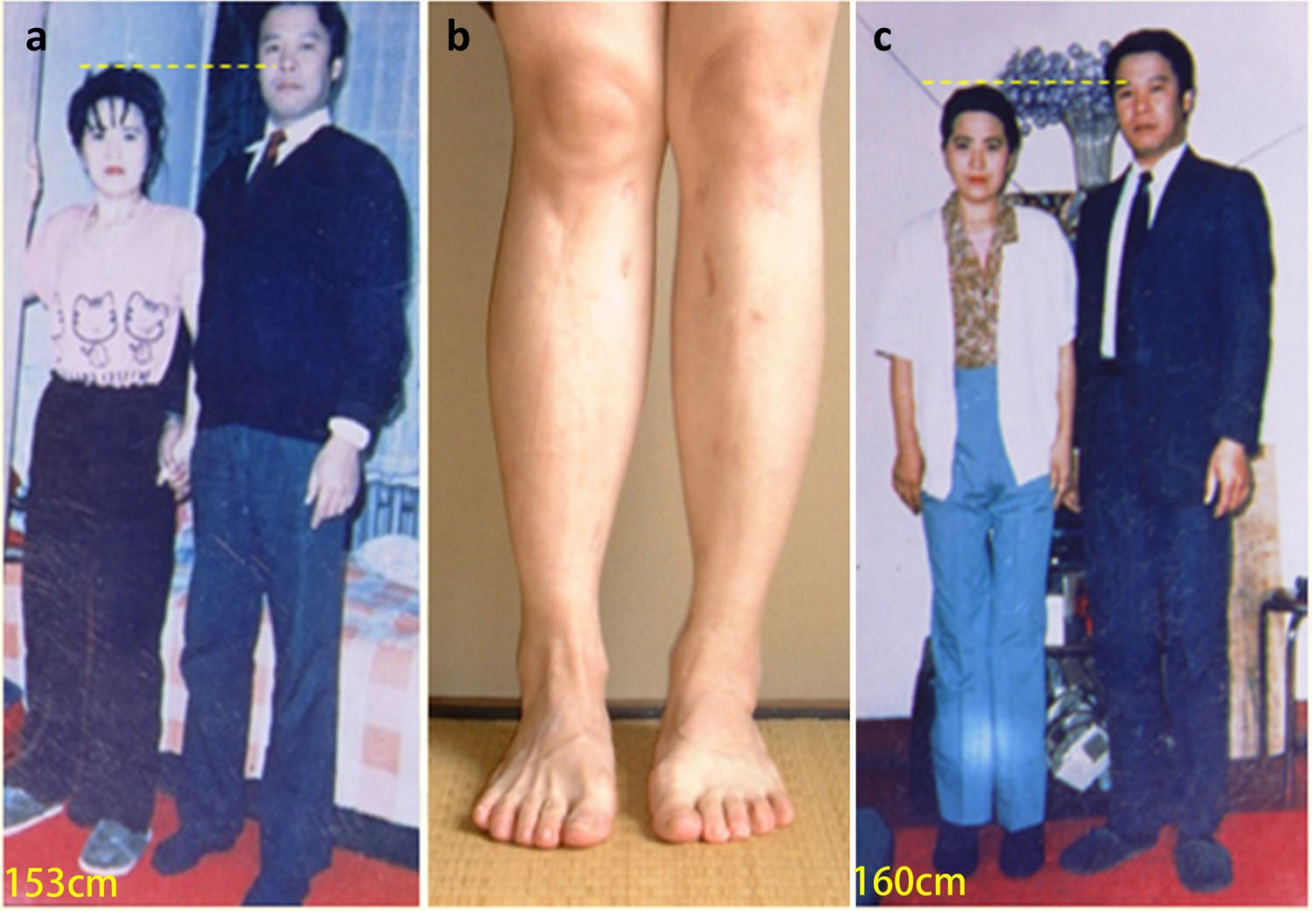
Yan-Sheng Wang and his wife. Before lengthening, his wife was 153 cm high (**a**), and after the lengthening of the legs (**b**), she was 7 cm (**c**). Provided by Dr. Long Qu

In 1993, Yan-Sheng Wang of Harbin finished the first limb lengthening surgery in China and the patient was his wife (Fig. 3). He performed a lot of bone lengthening surgeries but eventually abandoned them due to some lawsuits from his patients.

Long Qu, who studied under Kurosawa Takahide at Tokyo University, returned in 1997 and firstly used tibial transverse transposition to treat thromboangiitis obliterans and tried skull transverse transposition for craniocerebral diseases [8].

**Fig. 4:**
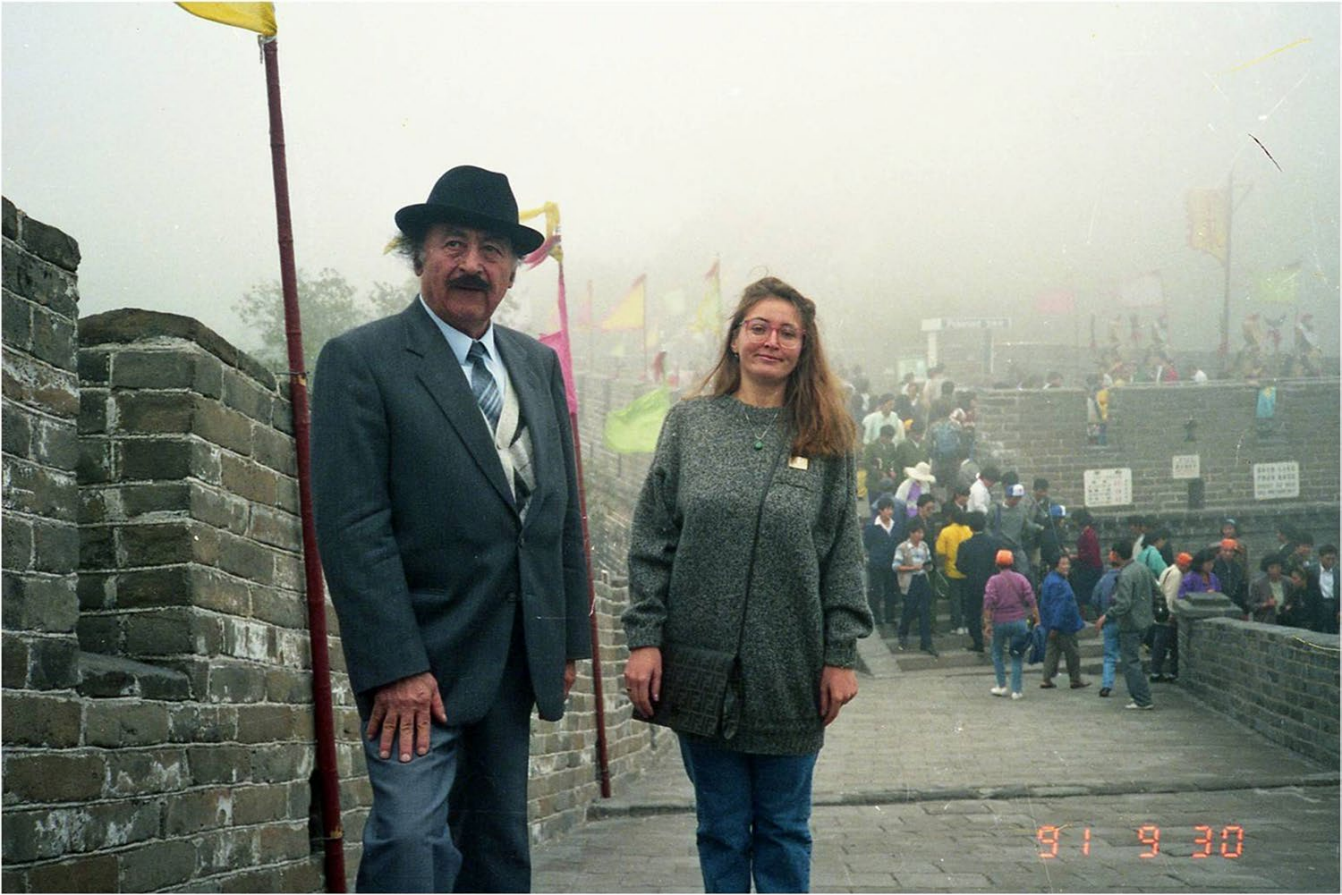
Ilizarov and his translator Irina Saranskikh (Иpинa Capaнcкиx) on the Great Wall

In 1991, Ilizarov was invited to the General Hospital of People’s Liberation Army in Beijing for an academic speech. The report lasted for four hours, with more than 500 slides. Ilizarov’s report became the driving force and catalyst for the development of his technology. This was the only time he visited China (Fig. 4). He passed away in 1992.

With the help of Soviet experts, Jian-Xin Yu of Jinzhou established the first Chinese Ilizarov Medical Center in 1992 [9]. Xiang-Sheng Zhang of Changsha learned Ilizarov bone lengthening in Italy. He developed an inlaid external fixator on callus lengthening for osteomyelitis and bone defects in children [10].

**Fig. 5:**
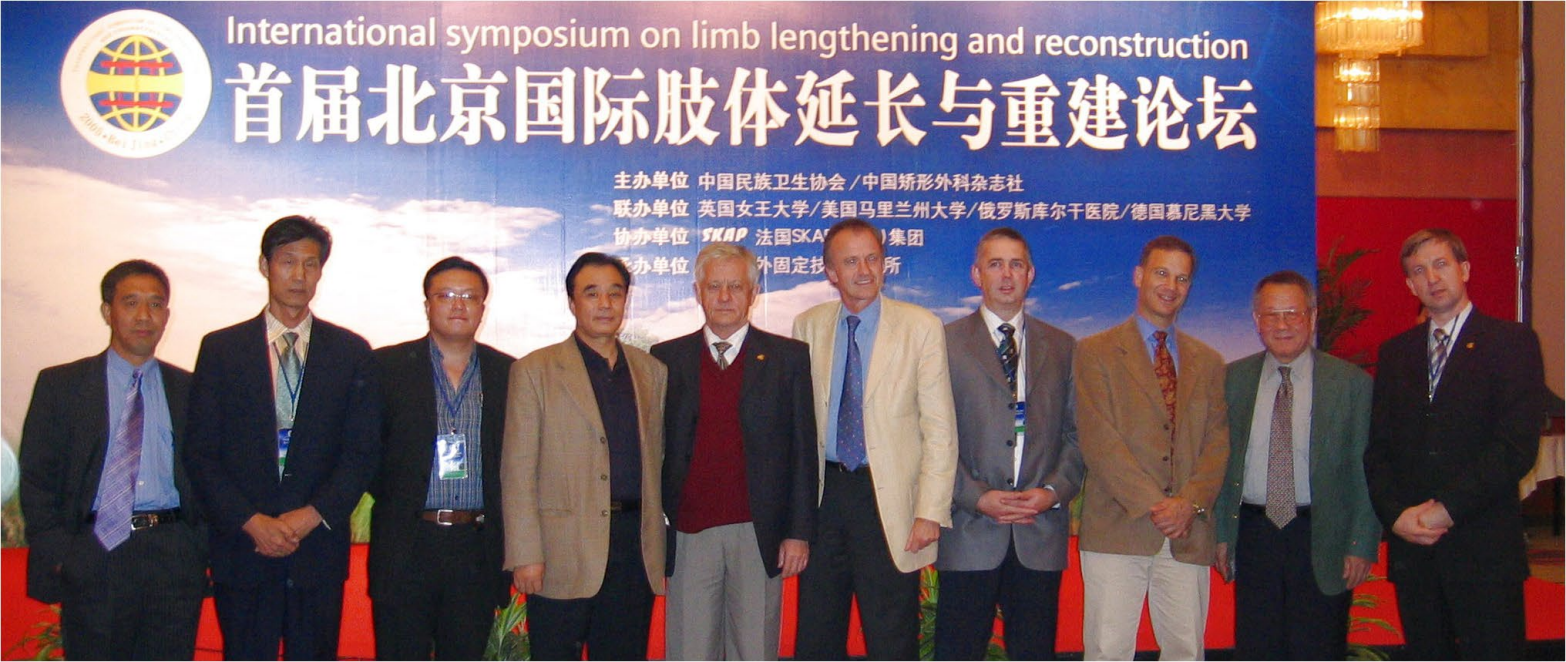
The first Beijing International Forum on limb lengthening and reconstruction. From left to right: Lang Yang, Si-He Qin, Gang Li, Tao-He Xia, VIadimir I Shevtsonv, Rainer Baumgart, Michael Liverick, Dror Paley, Yisu Zhao, Novikov Konstantin

In 2005, He-Tao Xia established the *Beijing Institute of External Fixation Technology* and the first specialized hospital of external fixation. *The First Beijing International Forum on limb lengthening and reconstruction* was attended by many famous international scholars including Shevstov and Paley (Fig. 5). This was the first large-scale international conference on Ilizarov technology in China.

In 2006, Si-He Qin, He-Tao Xia, and Gang Li went to Kurgan (Russia) and signed a cooperation agreement with Shevstov. In 2012, *the International Conference on External Fixation and Joint Reconstruction* was held in Beijing. With 506 participants, this is the largest Ilizarov Conference in China [11]. In 2013, Qin initiated the establishment of the *Chinese External Fixation Society (CEFS).*

## Second-generation members

In later years, Ilizarov technology is booming in China. A large number of scholars have emerged from all over the country [12]. Heng-Sheng Shu of Tianjin visited HSS hospital in 2004 and studied under Rozbruch. He introduced and carried out many Taylor Spatial Fixator surgeries [13]. In 2005, Xiu-Zhi Ren went to Baltimore of Maryland and studied in Paley’s Institute for one month. He began to use Ilizarov external fixation for osteogenesis imperfecta [14]. Since 2005, Lei Huang of Beijing began to use Orthofix’s external fixation for limb reconstruction [15]. In 2009, Qing-Lin Kang of Shanghai translated the book of Solomin [16].

## Modifications of instruments and technology

For years, Chinese surgeons have made a series of innovations of the instruments, surgical techniques, and treatment theory.

**Fig. 6:**
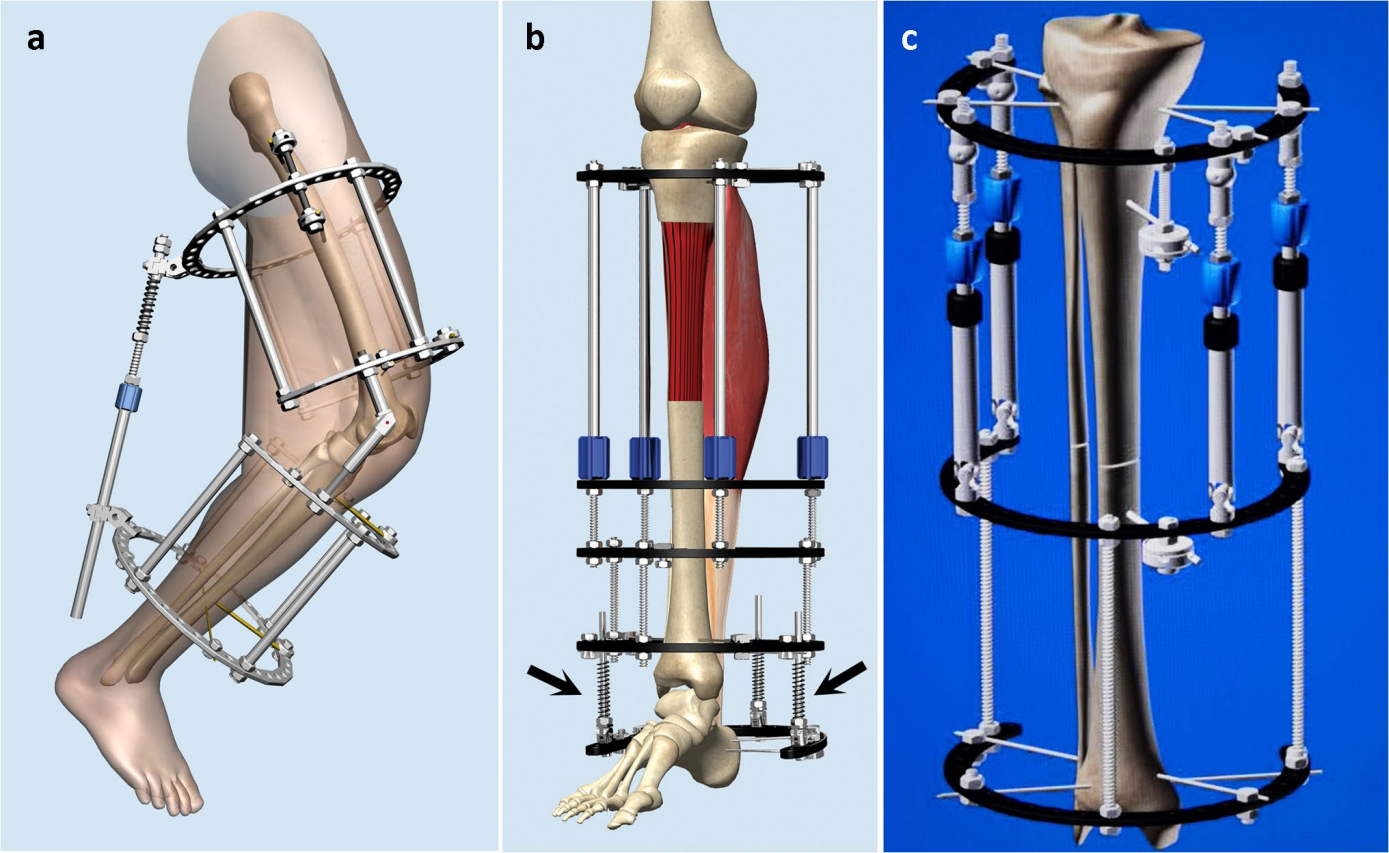
He-Tao Xia’s modifications on Ilizarov fixation: configuration for flexed knee deformity (**a**); configuration of leg lengthening and synchronization device of the tibia and Achilles tendon (the arrow) (**b**); double telescope rods design for limb lengthening (**c**)

**Fig. 7:**
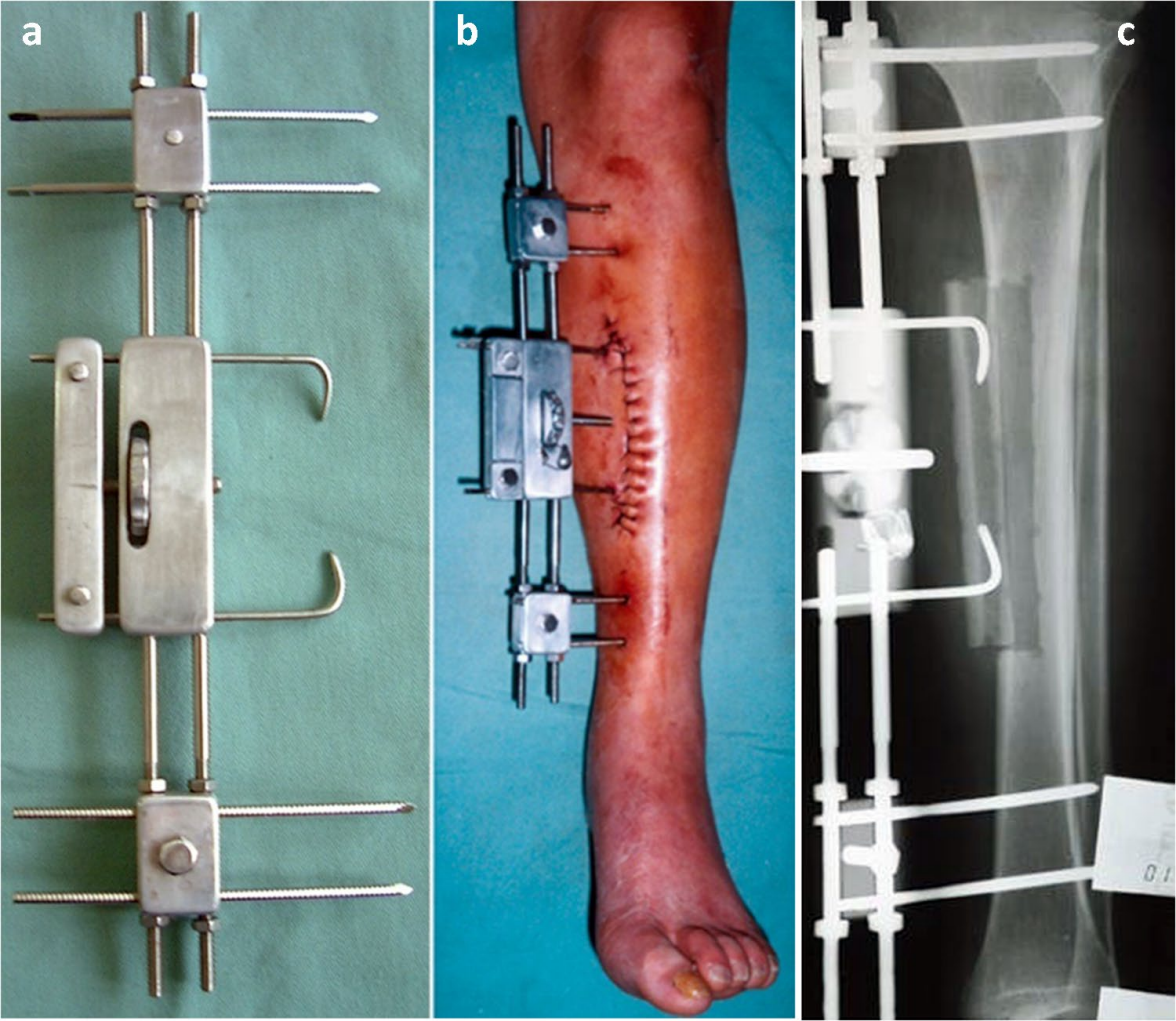
Transverse tibial transport instrument designed by Long Qu (**a**); the device was used for a case of thromboangiitis obliterans (**b**); X-film showed the transport was in the process (**c**)

**Fig. 8:**
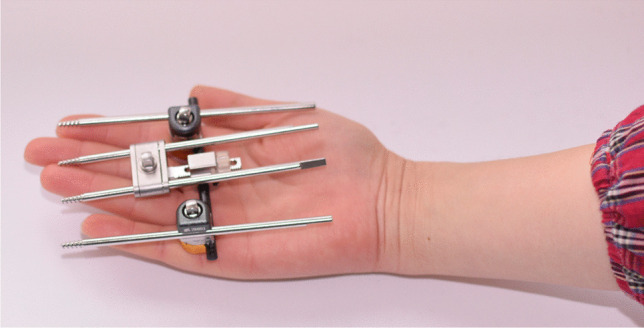
A mini-transverse tibial transport fixator designed by Yue-Liang Zhu

**Fig. 9:**
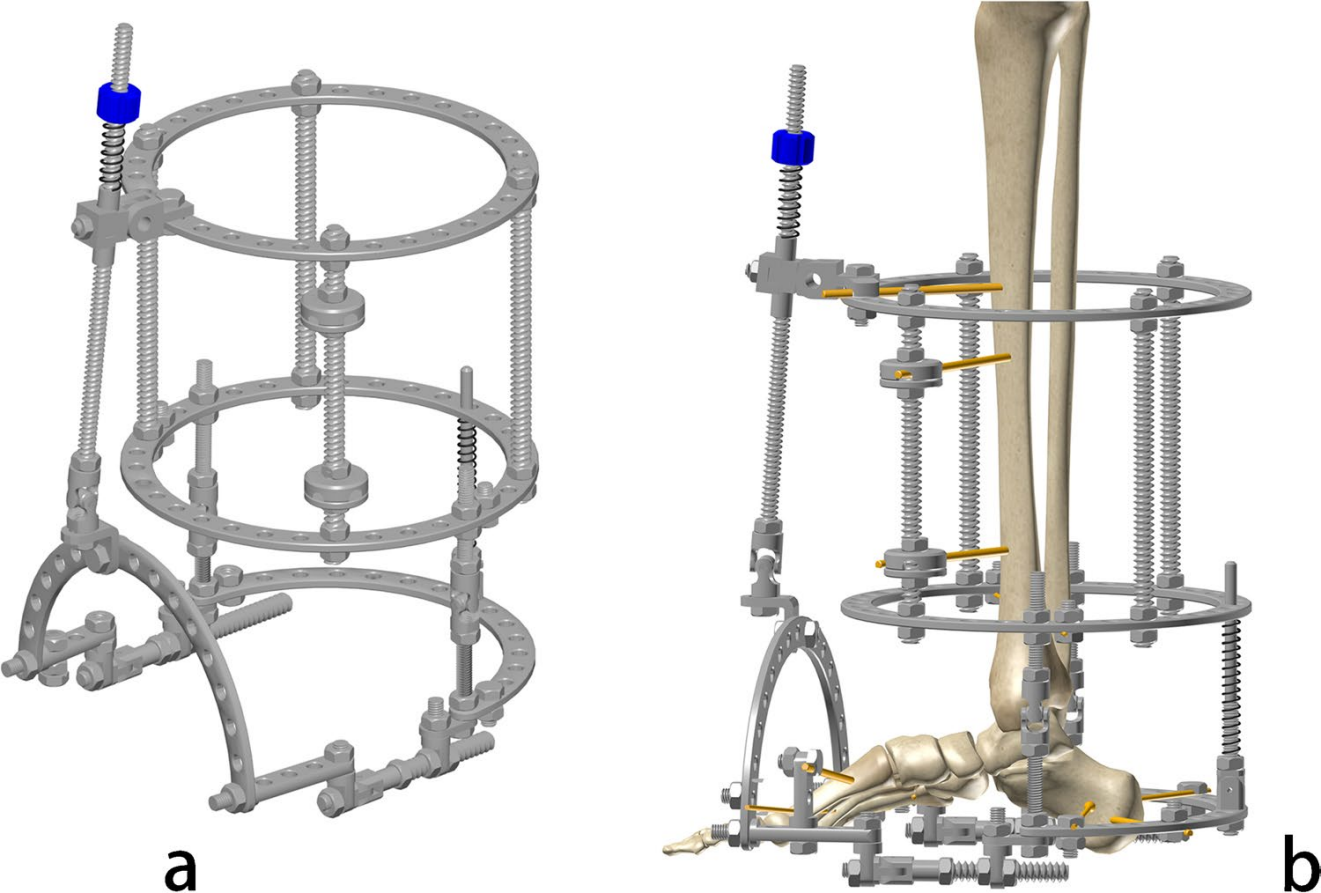
Universal configuration for foot and ankle deformities: oblique view (**a**) and lateral view (**b**)

1. The Sinicization of Ilizarov fixators (by He-Tao Xia): Xia modified the configuration and pinning of the fixators for “minimal components and maximal efficiency” [17] (Fig. 6).
2. Tendon balancing techniques (by Si-He Qin): Qin developed a set of tendons balancing techniques [1] to double the efficacy of Ilizarov external fixation and half the treatment time.
3. Synchronization device of the tibia and Achilles tendon (by He-Tao Xia and Si-He Qin): The device is widely used in leg lengthening to prevent foot drop [17, 18] (Fig. 6).
4. Transverse tibia transport (by Long Qu): Qu and other Chinese scholars have used this technique for thromboangiitis obliterans and diabetic foot with remarkable treatment outcomes (Fig. 7) [19–23]. Various fixators and instruments for TTT were invented and applied in recent years (Fig. 8). Their experience was shared at *the Fourth Combined Congress of ASAMI and ILLRS* at Liverpool. A consensus was reached on TTT for a diabetic foot in China [24].
5. Harbin phenomenon of bone transport (by Long Qu): In the process of bone transport, bone membrane and soft tissue in the defect site (not the osteotomy site) turned to be “new bone.” Qu named this “Harbin Phenomenon” [25].
6. Universal configuration for foot and ankle deformities (Xue-Jian Zheng): This configuration was widely used in China [26] (Fig. 9).

## Paper publication

**Fig. 10:**
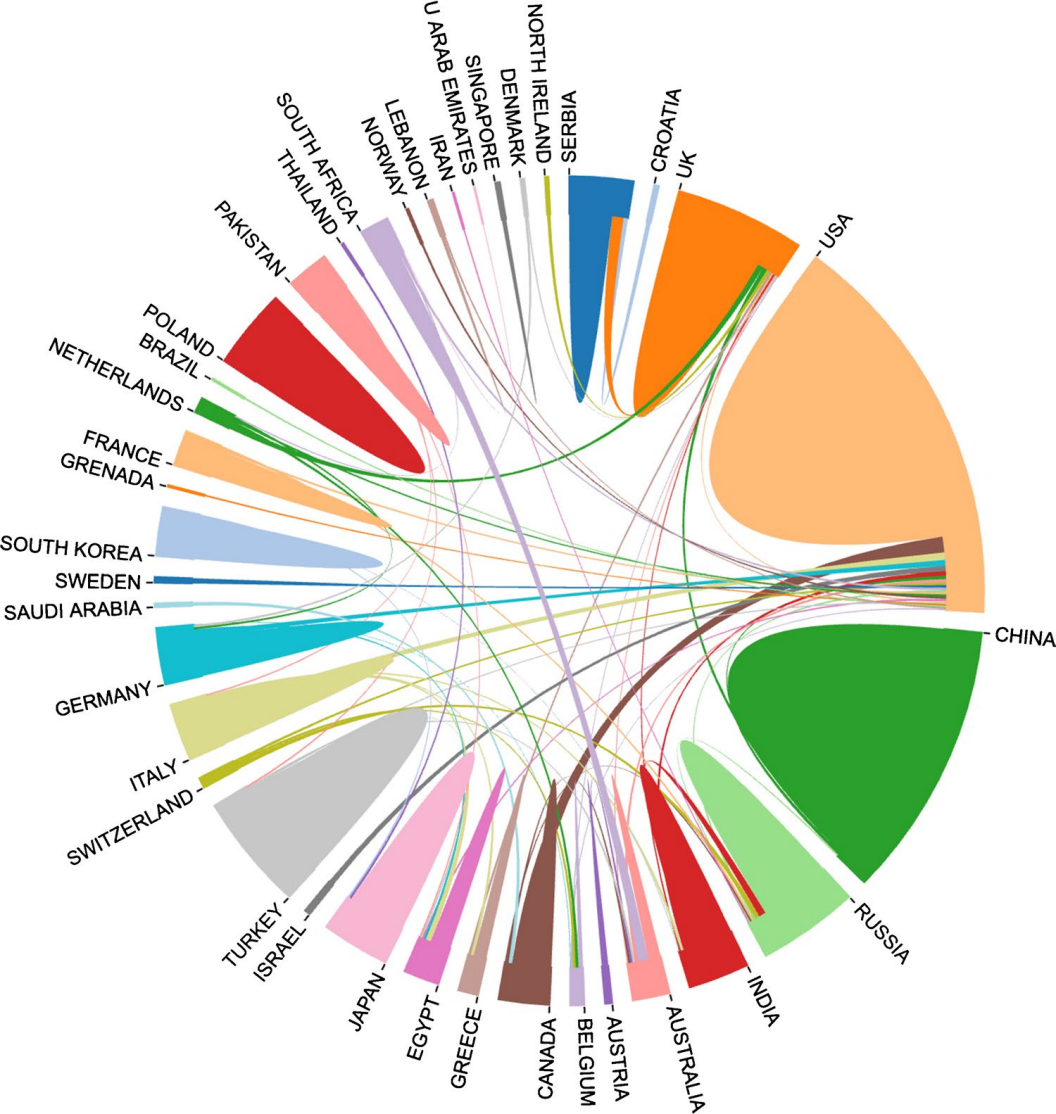
The schematic diagram of cooperative research relationship between countries and regions. Different colors represent different countries/regions. The countries/regions which have a larger area means they have more publications. The links indicate the international cooperation of researchers from different countries/regions

From 1986 to 2020, there were already published 1789 Chinese papers on Ilizarov techniques in 256 Chinese journals and 1709 English papers in 325 journals in the world. The top three countries were the USA (408), the UK (158), and China (121) [27]. In terms of literature quantity, China is only one quarter of that of the USA, ranking third. Within this literature, the USA has the highest proportion of total literature and more countries are involved in international cooperation, while Chinese scholars have only cooperated with the scholars of the USA, UKA, and Russia (Fig. 10).

## Conclusion

In the past 31 years, Ilizarov technology has taken root and spread extensively in China. Chinese scholars had many experiences and made modifications to this technology which is worthy to share with the world.

## Data Availability

Not applicable.
